# Differential surface density and modulatory effects of presynaptic GABA_B_ receptors in hippocampal cholecystokinin and parvalbumin basket cells

**DOI:** 10.1007/s00429-017-1427-x

**Published:** 2017-05-02

**Authors:** Sam A. Booker, Daniel Althof, Claudius E. Degro, Masahiko Watanabe, Ákos Kulik, Imre Vida

**Affiliations:** 1 Institute for Integrative Neuroanatomy, Neurocure Cluster of Excellence, Charité-Universitätmedizin Berlin, 10115 Berlin, Germany; 2 Institute of Physiology, University of Freiburg, Freiburg, Germany; 3 Department of Anatomy, Graduate School of Medicine, Hokkaido University, Sapporo, 0608638 Japan; 4 BIOSS Centre for Biological Signalling Studies, University of Freiburg, Freiburg, Germany; 5 Centre for Integrative Physiology, University of Edinburgh, Hugh Robson Building, George Square, Edinburgh, EH8 9XE UK

**Keywords:** Presynaptic inhibition, Synaptic transmission, GABAergic interneurons, Short-term plasticity, Electron microscope

## Abstract

The perisomatic domain of cortical neurons is under the control of two major GABAergic inhibitory interneuron types: regular-spiking cholecystokinin (CCK) basket cells (BCs) and fast-spiking parvalbumin (PV) BCs. CCK and PV BCs are different not only in their intrinsic physiological, anatomical and molecular characteristics, but also in their presynaptic modulation of their synaptic output. Most GABAergic terminals are known to contain GABA_B_ receptors (GABA_B_R), but their role in presynaptic inhibition and surface expression have not been comparatively characterized in the two BC types. To address this, we performed whole-cell recordings from CCK and PV BCs and postsynaptic pyramidal cells (PCs), as well as freeze-fracture replica-based quantitative immunogold electron microscopy of their synapses in the rat hippocampal CA1 area. Our results demonstrate that while both CCK and PV BCs contain functional presynaptic GABA_B_Rs, their modulatory effects and relative abundance are markedly different at these two synapses: GABA release is dramatically inhibited by the agonist baclofen at CCK BC synapses, whereas a moderate reduction in inhibitory transmission is observed at PV BC synapses. Furthermore, GABA_B_R activation has divergent effects on synaptic dynamics: paired-pulse depression (PPD) is enhanced at CCK BC synapses, but abolished at PV BC synapses. Consistent with the quantitative differences in presynaptic inhibition, virtually all CCK BC terminals were found to contain GABA_B_Rs at high densities, but only 40% of PV BC axon terminals contain GABA_B_Rs at detectable levels. These findings add to an increasing list of differences between these two interneuron types, with implications for their network functions.

**Electronic supplementary material:**

The online version of this article (doi:10.1007/s00429-017-1427-x) contains supplementary material, which is available to authorized users.

## Introduction

The perisomatic domain of cortical pyramidal cells (PC) is under GABAergic control of a specific subset of inhibitory interneurons called basket cells (BCs) (Buhl et al. 1994; Freund and Buzsáki 1996; Klausberger and Somogyi 2008). BCs are heterogeneous, comprising two main types with distinct neurochemical and anatomical characteristics, which are believed to underlie their divergent roles in functional networks. The two BC types are primarily distinguished on the basis of their selective expression of either the neuropeptide cholecystokinin (CCK) or the calcium-binding protein parvalbumin (PV) (Nunzi et al. 1985; Kosaka et al. 1987; Freund and Katona 2007; Lee and Soltesz 2011; Booker et al. 2013, 2017). Furthermore, CCK and PV BCs also differ in their physiological properties, most prominently their regular- versus fast-spiking discharge pattern, respectively.

Action potential-evoked release of GABA from inhibitory terminals, including those of BCs, is dependent on reliable activation of high voltage-gated calcium channels (VGCCs) leading to vesicular GABA release, which is tightly controlled by presynaptic neuromodulatory receptors for multiple transmitter systems (Wu and Saggau 1995; Lüscher et al. 1997; Lee and Soltesz 2011). The two BC types show striking differences in presynaptic mechanisms as well: in axon terminals of CCK BCs Ca_v_2.2 (N-type) VGCCs are localized to the active zone, while in PV BCs Ca_v_2.1 (P/Q-type) channels are present (Hefft and Jonas 2005; Lee and Soltesz 2011). Moreover, the compliment of presynaptic neuromodulatory receptors also diverges: CCK BCs possess high levels of endocannabinoid 1 receptors (CB1Rs), whilst PV BCs possess muscarinic acetylcholine 2 (M2Rs) and µ-opioid receptors (Hajos et al. 1997; Katona et al. 1999; Fukudome et al. 2004; Neu et al. 2007; Armstrong and Soltesz 2012; Lenkey et al. 2015). Despite these differences, both CCK and PV BCs appear to express GABA_B_ autoreceptors at their output synapses (Lee and Soltesz 2011; Booker et al. 2013).

GABA_B_Rs are heterodimeric receptors composed of the GABA_B1_ and GABA_B2_ subunits (Kaupmann et al. 1998). At presynaptic membranes GABA_B_Rs comprise the Sushi-domain containing GABA_B1a_ splice variant (Guetg et al. 2009) and negatively couple to VGCCs via the G-protein (G_i/o_) βγ subunit. Accordingly, activation of presynaptic GABA_B_Rs by the canonical agonist baclofen has been shown to result in a reduction of unitary IPSC amplitudes at both CCK and PV BC synapses (Hefft et al. 2002; Lee and Soltesz 2011; Booker et al. 2013; Jappy et al. 2016). However, direct comparison is difficult due to large differences in the concentration of agonist applied and recording conditions in the individual studies. Furthermore, the subcellular localization and density of the GABA_B_Rs in synaptic and extrasynaptic membrane segments of CCK and PV BC axon terminals has not yet been investigated.

In this study therefore we comparatively assessed the function and localization of presynaptic GABA_B_Rs in identified BC axon terminals, using whole-cell patch-clamp recordings and quantitative SDS-digested freeze-fracture replica (SDS-FRL) immunoelectron microscopy.

## Materials and methods

### Animals

All experiments were performed on 17–26-day-old rats, wild-type or transgenic expressing Venus/yellow fluorescence protein (YFP) under the vesicular GABA transporter (vGAT) promoter, both on the Wistar background (Uematsu et al. 2008; Booker et al. 2014, 2017). SDS-FRL electron microscopic analysis was performed on 60-day-old wild-type Wistar rats (2 rats). All experiments were performed in accordance with institutional (Charité-Universitätmedizin Berlin; University of Freiburg, Freiburg, Germany), local (LaGeSo, Berlin, T 0215/11), and national guidelines (German Animal Welfare Act; ASPA, United Kingdom Home Office).

### Acute slice preparation

Hippocampal slices were prepared as previously described (Booker et al. 2014). Briefly, rats were decapitated, the brain rapidly removed and placed in ice-cold carbogenated (95% O_2_/5% CO_2_) sucrose-modified artificial cerebrospinal fluid (in mM: 87 NaCl, 2.5 KCl, 25 NaHCO_3_, 1.25 NaH_2_PO_4_, 25 glucose, 75 sucrose, 7 MgCl_2_, 0.5 CaCl_2_, 1 Na-pyruvate, 1 Na-ascorbate). Transverse, 300 μm thick slices were then cut on a Vibratome (VT1200 s, Leica, Germany), stored submerged in sucrose-ACSF warmed to 35 °C for 30 min and subsequently at room temperature until the start of recording.

### Whole-cell patch-clamp recordings

For whole-cell patch-clamp recordings, slices were transferred to a submerged recording chamber perfused with carbogenated ACSF (in mM: 125 NaCl, 2.5 KCl, 25 NaHCO_3_, 1.25 NaH_2_PO_4_, 25 glucose, 1 MgCl_2_, 2 CaCl_2_, 1 Na-pyruvate, 1 Na-ascorbate) maintained at near physiological temperatures (32 ± 1 °C) with an inline heater (SuperTech, Switzerland) at a high flow rate of 10–12 ml min^−1^. Slices were visualized with infrared differential contrast illumination by means of an upright microscope (BX-50, Olympus, Hamburg, Germany) equipped with a 40× water-immersion objective (N.A. 0.8). BCs were pre-selected for recording based on vGAT-Venus fluorescence, by means of epifluorescent illumination (480 nm), in large somata in and around *str. pyramidale* and proximal *str. radiatum.* Whole-cell patch-clamp recordings were accomplished using either an AxoPatch 200B or Multiclamp 700B amplifier (Molecular Devices, USA). Recording pipettes were pulled from borosilicate glass capillaries (2 mm outer/1 mm inner diameter, Hilgenberg, Germany) on a horizontal electrode puller (P-97, Sutter Instruments, CA, USA). When filled with intracellular solution [composition in mM: 100 K-Gluc, 40 KCl, 2 MgCl_2_, 10 EGTA, 10 HEPES, 2 Na_2_-ATP, 0.3 Na_2_-GTP, 1 Na_2_-Creatinine, 0.1% biotinylated-lysine (Biocytin, Invitrogen, UK); pH: 7.4, Osmolarity: 280–300 mOsm] a pipette resistance of 3–5 MΩ was achieved with a Cl^−^ reversal potential (*E*_R_) of ~30 mV. Unless otherwise stated, all voltage-clamp recordings were performed at −65 mV and all current-clamp recordings from the resting membrane potential (*V*_M_). Throughout the recordings, the series resistance was monitored, but not compensated. Signals were filtered at 10 kHz using the built in 4-pole Bessel filter of the amplifier, digitized at 20 kHz on an analog–digital interface (CED 1401, Cambridge Instruments, Cambridge, UK; or NI USB-6212 BNC, National Instruments, Berkshire, UK), and acquired with WinWCP software (courtesy of John Dempster, Strathclyde University, Glasgow, UK; http://spider.science.strath.ac.uk/sipbs/software_ses.htm). Data was analyzed offline using the open source Stimfit software package (Guzman et al. 2014).

### Characterization of presynaptic GABA_B_R function

Following breakthrough into whole-cell configuration, intrinsic properties of recorded neurons were characterized in current-clamp mode from resting *V*_M_. Neurons were physiologically identified based on their response to hyper- to depolarizing current steps (−500 to 500 pA, in 100 pA steps, 500 ms duration). CCK BCs were identified as responding to depolarizing stimulus with low to moderate frequency trains of action potentials (APs); PV BCs were identified as firing high-frequency non-accommodating trains (>100 Hz) of APs. PCs showed characteristic large amplitude and broad APs, and lower-frequency accommodating trains of APs. Neurons were rejected from further analysis if *V*_M_ was more depolarized than −50 mV, APs did not overshoot 0 mV, or initial series resistance exceeded 30 MΩ or changed by >20% over the course of the experiment.

Minimally stimulated monosynaptic inhibitory post-synaptic currents (IPSC) in PCs were elicited in the presence of NBQX (10 µM) and APV (50 µM) by extracellular stimulation with a glass monopolar electrode (a patch pipette filled with 2 M NaCl) placed in *str. pyramidale* at a distance of 10–20 µm from the soma. IPSC were evoked every 10 s with stimulus amplitudes of 1–20 V, minimally exceeding threshold to evoke a response. A 5-min control baseline was collected after which 1 µM WIN-55,212 (WIN) was briefly applied to the bath (2 min) to test presynaptic CB1R sensitivity. Following washout of WIN and recovery of IPSC amplitude to control levels (~5 min) baclofen (10 μM) was applied to the bath and steady state inhibition was observed at 3–5 min following wash in. Following baclofen application, CGP-55,845 (CGP, 5 μM) was applied to the bath confirming that the observed responses were GABA_B_R mediated. In recordings where the IPSC was not sensitive to WIN application, we subsequently applied the selective M2R agonist arecaidine but-2-ynyl ester tosylate (ABET; 10 µM) (Chiang et al. 2010) to the bath to confirm that these WIN-insensitive IPSCs were mediated by M2R-containing putative PV axon terminals. In all recordings the whole-cell holding current (*I*_WC_) in the CA1 PC was monitored to confirm successful activation of postsynaptic GABA_B_Rs, given that presynaptic receptors show higher baclofen affinity (Dugladze et al. 2013). Given the optimal voltage-clamp conditions for the recording of perisomatic synaptic events meditated by BCs, we did not block post-synaptic Kir3 potassium channels in any of these experiments.

To confirm that presynaptic GABA_B_R mediated effects were specific to identified BCs, we performed paired recordings between a CCK or PV BC and a CA1 PC. Conditions were identical to those of single cell recordings albeit with a lower 0.5 mM EGTA in the intracellular solution (compensated by a higher K-gluconate content of 110 mM). Following characterization of intrinsic properties (see above) of pre- and postsynaptic neurons, 10 trains of 10 APs (1–2 nA, 1 ms, 20 Hz) were elicited in the BC while recording the CA1 PC in voltage-clamp (as above). If a unitary synaptic connection was observed, seen as short-latency (<4 ms) IPSCs in the PC following presynaptic APs in averages of ten traces, it was assumed that there was a direct synaptic connection. If synaptic connectivity was not observed in the CA1 PC, the recording was abandoned, an outside-out patch formed, and a neighboring CA1 PC recorded. Once a synaptic connection was found we recorded >50 traces under paired-pulse protocols (2 stimuli with 50 ms interval), following which baclofen (10 μM) was bath applied for 5 min, after which CGP (5 μM) was bath applied. The IPSC amplitude was measured from the preceding baseline as an average over a 0.4 ms window corresponding to the peak within 10 ms from the start of the AP. Onset latency of the IPSC was assessed as the duration from the time of maximal rise rate of the presynaptic AP to the onset of the IPSC. Mean IPSC responses shown and measured from at least 30 traces.

It should be noted that we have included reanalyzed data (7 pairs) from a previous publication (Booker et al. 2013) where we had measured and reported only the unitary IPSC amplitudes for PV BC connections. We have assessed the presynaptic properties of those synaptic connections together with a set of newly recorded pairs (4 pairs) under identical conditions.

### Visualization, imaging and reconstruction of the recorded neurons

Post hoc identification was performed on all recorded neurons as previously described (Booker et al. 2013, 2014, 2017). Briefly, following successful outside-out patch formation, slices were fixed in 4% paraformaldehyde (PFA) in 0.1 M phosphate buffer (PB) overnight at 4 °C. Slices were washed in PB and phosphate buffered saline (PBS; 0.025 M PB + 0.9% NaCl), then blocked with 10% normal goat serum (NGS), 0.3–0.5% TritonX-100, and 0.05% NaN_3_ all diluted in PBS at room temperature (RT) for 1 h. Slices were then incubated in primary antibodies for 48–72 h in a solution containing 5% NGS, 0.3–0.5% TritonX-100 and 0.05% NaN_3_in PBS at 4 °C. CCK BCs were identified with a primary antibody recognizing CCK (Mouse monoclonal 1:5000, G. Ohning, CURE, UCLA, CA, USA). PV BCs were identified using a primary antibody against PV (mouse monoclonal, SWANT, Marly, Switzerland; 1:5000; see Booker et al. 2013). Slices were washed in PBS and then incubated with anti-mouse fluorescent secondary antibodies (goat anti-mouse Alexa Fluor 546, 1:500; Invitrogen, Dunfermline, UK) with fluorescently conjugated streptavidin (Alexa Fluor 647; 1:500, Invitrogen) in a solution containing 3% NGS, 0.1% TritonX-100 and 0.05% NaN_3_ at 4 °C overnight (O/N). Slices were then washed in PBS, then PB, and mounted on glass slides with a 300 µm agar spacer, with a polymerizing mounting medium (Fluoromount-G, Southern Biotech, AL, USA) and coverslipped.

Neurons were imaged using a confocal laser scanning microscope (FluoView 1000, Olympus) with a 20× (N.A. 0.75) lens and *z*-axis stacks of images (4 Mpixel resolution, 1 μm *z*-steps) collected to allow identification of somatodendritic and axonal arborizations. Immunoreactivity of recorded neurons was tested at the soma with a silicon-immersion 60× (N.A. 1.3) objective lens. Neurons were reconstructed offline from digitally stitched image stacks, which were segmented and reconstructed using semi-automatic analysis software (Simple Neurite Tracer plug-in for the ImageJ/FIJI software package; http://fiji.org; Longair et al. 2011).

### Antibody characterization for immunogold labeling

The specificity of the affinity-purified antibodies to CB1R (Fukudome et al. 2004), M2R (Alomone Labs, Israel), GABA_B1_ (B17, Kulik et al. 2002; B62, Kulik et al. 2006) were assessed with immunoblot analysis of crude membrane fractions derived from either mouse brains (CB1R and M2R) or rat brains (B17 and B62). Single protein bands were detected with M2R and CB1R antibodies, whereas GABA_B1_ antibodies gave rise to two immunoreactive products with estimated molecular masses of 130 and 100 kDa, as expected from the two splice variants of the GABA_B1_ subunit. In addition the band for M2R was not detected in M2R knock-out brains (data not shown). All antibodies used targeted the intracellular epitopes of the transmembrane proteins and therefore resulted in specific labelling on the protoplasmic face (P-face) of the replicas.

### SDS-digested freeze-fracture replica immunogold labeling (SDS-FRL)

SDS-FRL was performed as previously described (Althof et al. 2015). Briefly, 60-day-old Wistar rats (2) were perfusion fixed with 2% PFA and 15% saturated picric acid in 0.1 M PB. Transverse 100 μm hippocampal sections were cut and cryoprotected O/N in PB with 30% glycerol at 4 °C. Blocks containing the *str. pyramidale and radiatum* of the CA1 area were blocked-out and then frozen by a high-pressure freezing machine (HPM100, Leica, Austria). Frozen samples were freeze-fractured at −140 °C and coated by deposition with carbon (5 nm), platinum (2 nm) and carbon (18 nm) in a freeze-fracture replica device (BAF 060, BAL-TEC, Lichtenstein). Replicas were digested for 18 h at 80 °C in a solution containing 2.5% SDS and 20% sucrose in 15 mM Tris buffered saline (TBS) at pH 8.3. Replicas were then rinsed in washing buffer [0.05% bovine serum albumin, (BSA) and 0.1% Tween 20, in TBS] and blocked in a solution containing 5% BSA and 0.1% Tween 20 for 1 h at RT. Subsequently, replicas were incubated in one of the following mixtures of primary antibodies: (a) CB1R [Guinea pig (Gp); 1:3000, Fukudome et al. 2004] and GABA_B1_ [B17, Rabbit (Rb); 5 µg/ml, Kulik et al. 2002], (b) M2R (Rb; 10 µg/ml, Alomone Labs, Israel) and GABA_B1_ [B62, (Gp); 10 µg/ml, Kulik et al. 2006] made up in 50 mM TBS containing 1% BSA and 0.1% Tween 20 O/N at 4 °C. Replicas were then rinsed in TBS, blocked for 30 min and reacted with a mixture of either (a) gold-coupled 10 nm goat anti-guinea pig IgG and 5 nm goat anti-rabbit IgG or (b) 10 nm goat anti-rabbit IgG and 5 nm goat anti-guinea pig IgG secondary antibodies (1:30; BioCell Research Laboratories, Cardiff, UK) diluted in TBS containing 1% BSA and 0.1% Tween 20 for 3 h at RT or at 4 °C O/N. Replicas were subsequently rinsed in TBS, ultrapure water and then mounted on 100-mesh grids. Quantitative analysis was performed at 6000–10,000× magnification under an electron microscope (Philips CM100). Images were taken with a CCD camera (Orius SC600, GATAN, Inc.) and acquired with GATAN software. Presynaptic axon terminals were collected in *str. pyramidale* and identified as perisomatic due to their direct apposition to a somatic or very proximal dendritic membranes. Putative CA1 PC spiny dendrites were collected from *str. radiatum* as positive control. Immunogold particle density was calculated by counting the number of particles on the 2D surface of identified axon terminals and dendrites using the FIJI software package.

### Statistical analysis

Statistical analysis was performed with Graphpad Prism 3.0 (GraphPad Software, CA, USA). Group data were compared with one-way ANOVA tests, combined with Bonferroni post-test to establish group differences. Analysis of unpaired and paired data was performed with Mann–Whitney or Wilcoxon matched-pairs tests, respectively. Data are shown as mean ± SEM throughout. Statistical significance was assumed if *P* < 0.05.

## Results

### Presynaptic GABA_B_Rs differentially inhibit CB1R- and M2R-sensitive perisomatic inhibitory transmission

**Fig. 1:**
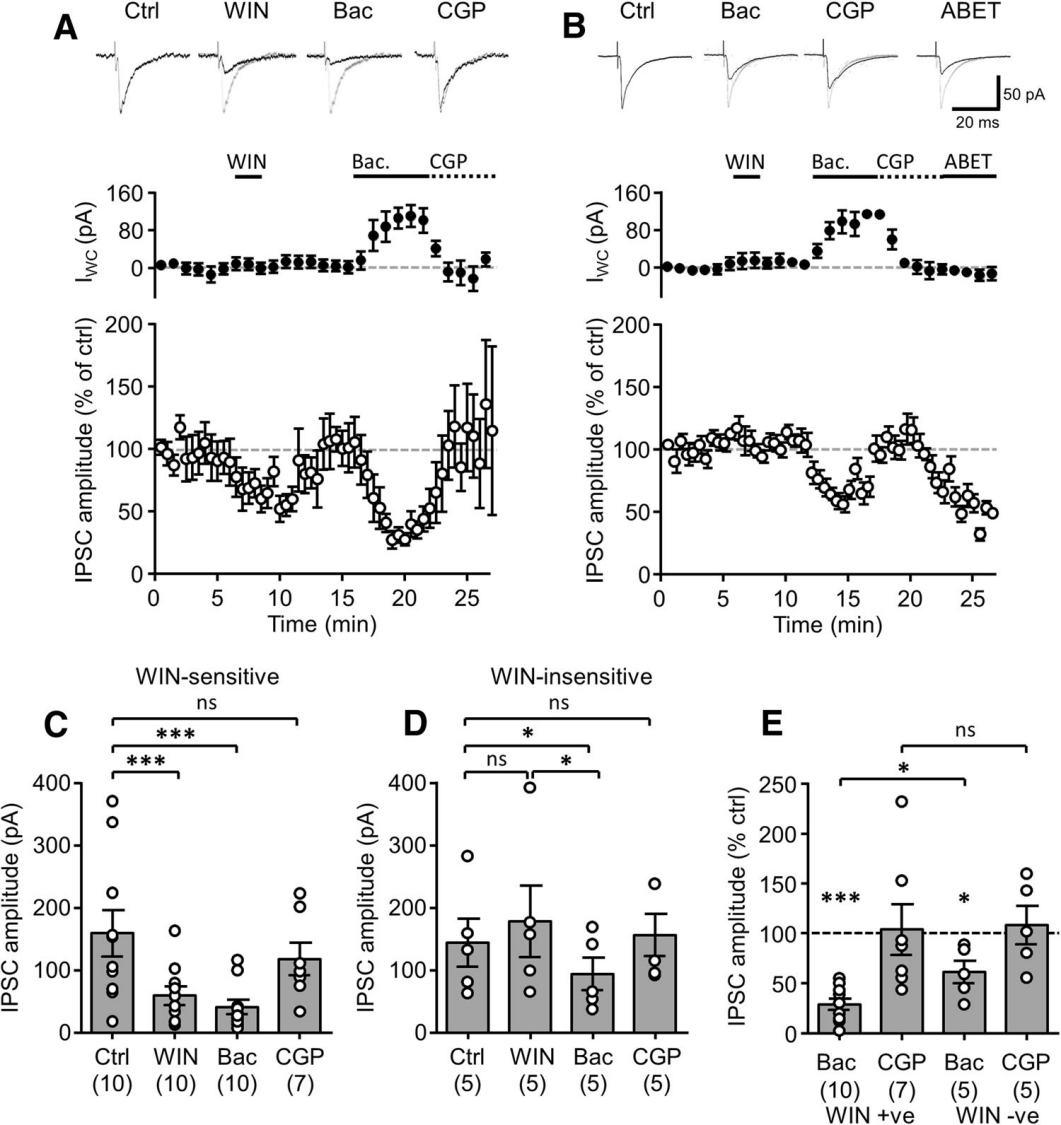
Presynaptic GABA_B_Rs inhibit transmission at CB1R-expressing perisomatic GABAergic terminals more strongly than M2R-positive terminals. **a** Representative WIN-sensitive IPSCs in a CA1 PC evoked by minimal stimulation delivered to the CA1 *str. pyramidale* (*top traces*) under control conditions (Ctrl) and during sequential bath application of WIN-55212 (WIN, 1 µM, 2 min), baclofen (Bac, 10 µM, 5 min) and CGP-55845 (CGP, 10 µM, 5 min); control trace is underlain for all conditions for comparison. Time-course plots of the mean *I*_WC_ (*middle*) and normalized amplitude of the WIN-sensitive IPSCs recorded from 10 CA1 PCs (*bottom*). **b** Effects of baclofen application on WIN-insensitive IPSCs presented as in **a**. These IPSCs were consistently inhibited by subsequent application of the selective M2R agonist, ABET (10 µM, 5 cells). *Summary bar charts* of the mean IPSC amplitudes elicited by WIN-sensitive (**c**) and insensitive afferents (**d**) measured at the end of each pharmacological epoch. Numbers of recorded PCs indicated in *brackets*. **e** *Bar chart* of the normalized IPSC amplitudes elicited by WIN-sensitive (WIN +ve) and insensitive afferents (WIN−ve) during Bac and CGP application. Statistics shown: *ns P* > 0.05, * P < 0.05, *** P < 0.001, Mann–Whitney or Wilcoxon signed rank tests, corrected for multiple comparisons

To assess the role of presynaptic GABA_B_Rs in pharmacologically identified putative CCK versus PV BC axon terminals, we first recorded monosynaptic IPSCs evoked by minimal stimulation from CA1 PCs (15 cells) in the presence of ionotropic glutamate receptor blockers (NBQX: 10 µM and APV: 50 µM). All CA1 PCs were morphologically identified, as previously described (Degro et al. 2015), and had a soma in *str. pyramidale* with a single large caliber apical dendrite projecting through *str. radiatum*, with all dendrites decorated with dendritic spines. Stimulation via electrodes placed in the *str. pyramidale,* in close proximity to the recorded PCs (10–20 µm distal) consistently produced fast IPSCs. To identify CB1R-sensitive responses, corresponding to putative CCK BC afferents, brief (2 min) bath application of the highly potent receptor agonist WIN-55212 (WIN; 1 µM) was performed. In ten cells, this brief application of WIN reduced the IPSC amplitude to 58.3 ± 15.7% (*P* = 0.002, Wilcoxon matched-pairs test, Fig. 1a) confirming the presence of CB1R on the stimulated axons in this subset of recordings. Following complete washout of WIN and full recovery of the IPSCs to control levels, the selective GABA_B_R agonist baclofen was bath applied (10 µM, 5 min) resulting in a large decrease in IPSC amplitude to 29.9 ± 5.5% of control (*P* = 0.0001, Wilcoxon matched-pairs test, Fig. 1a, c, e). In addition, there was an outward *I*_WC_ induced in the recorded PCs (95.6 ± 12.0 pA; *P* = 0.001) consistent with GABA_B_R-mediated activation of a postsynaptic potassium current (Booker et al. 2013, 2017; Fig. 1a, middle). Both the IPSC amplitude and *I*_WC_ were fully recovered to control levels with the subsequent removal of baclofen and application of the potent and selective GABA_B_R antagonist CGP-55845 (CGP; 5 µM).

In five recorded PCs, WIN application had no inhibitory effect on the IPSC amplitude (Fig. 1b). In these WIN-insensitive afferents, bath application of baclofen reduced the IPSC amplitude only to 61.5 ± 11.3% of control (*P* = 0.04), indicating an approximately twofold weaker inhibition than for WIN-sensitive afferents (*P* = 0.024, Mann–Whitney test, Fig. 1b, d, e). Similar to the recordings for CB1R-sensitive afferents, baclofen application induced a postsynaptic outward *I*_WC_ of 83.7 ± 18.3 pA in the postsynaptic PCs (compared to control: *P* = 0.02; CB1R-insensitive versus -sensitive: *P* = 0.75, Mann–Whitney test). To confirm that the CB1R-insensitive afferents corresponded to M2R-containing putative PV BC axons, we subsequently bath applied the selective agonist ABET (10 µM, 5 min). Indeed, in response to ABET application, IPSC amplitude was reduced in all five recorded PCs to 51.2 ± 14.4% of control. Neither WIN nor ABET application resulted in significant change in holding current of the recorded neurons, indicating a predominantly presynaptic locus of activity under these recording conditions (WIN: *P* = 0.89; ABET: *P* = 0.66; Wilcoxon signed rank tests). These data indicate that both populations of pharmacologically identified CA1 BC axon terminals contain functional GABA_B_Rs, but are differentially inhibited by these autoreceptors.

### Strong presynaptic GABA_B_R-mediated inhibition at the synaptic output of CCK BCs

**Fig. 2:**
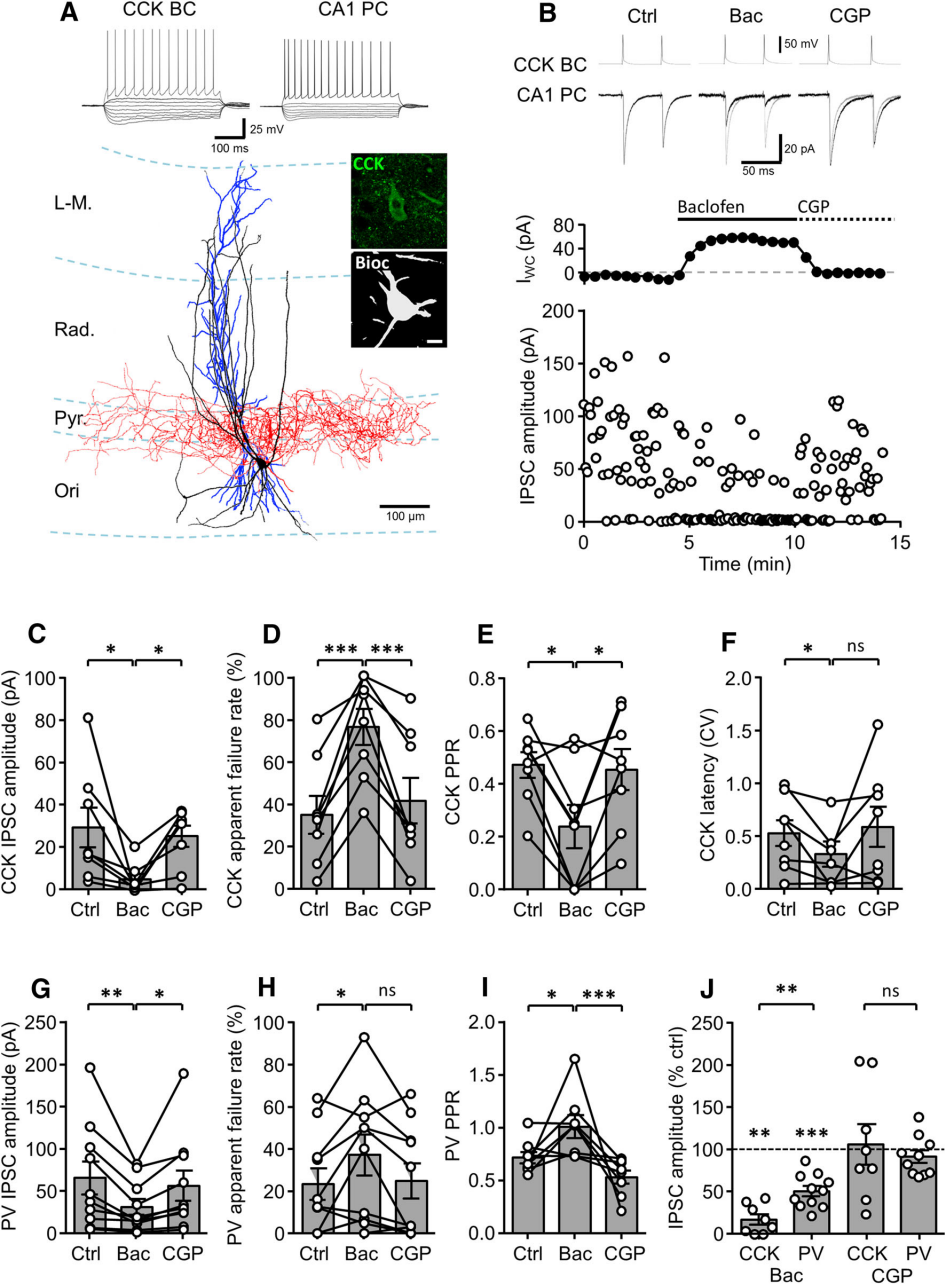
Unitary IPSCs produced in CA1 PCs by identified CCK and PV BCs show differential presynaptic GABA_B_R-mediated inhibition. **a** Reconstruction of a synaptically coupled CCK BC (soma and dendrites in *black*, axon in *red*) and CA1 PC (soma and dendrites in *blue*) pair. *Inset*, *top*, voltage responses elicited in the CCK BCs and the CA1 PCs to hyper- to depolarizing current steps (−500 to 500 pA, 50 pA steps, 500 ms duration). Note the regular-spiking phenotype of the CCK BCs. *Inset*, *right*, immunolabeling for CCK (in *green pseudocolor*) in the biocytin-filled soma of the BC (in *gray*). Pyr., *stratum pyramidale*; Ori., *stratum oriens*; Rad., *stratum radiatum*; L-M, *stratum lacunosum*-*moleculare*. **b** *upper*, representative pairs of action potentials elicited in the same CCK BC as in **a** (*upper traces*, suprathreshold current pulses of 1–2 nA, 1 ms duration, 50 ms interval) resulted in unitary IPSCs in the post-synaptic CA1 PC (*lower traces*) under control conditions (*left panel*), followed by sequential bath application of baclofen (10 µM, *middle panel*), and CGP (10 µM; *right panel*); baclofen and CGP traces are underlain by control traces (*gray*). **b** *lower*, Time-course plot of the postsynaptic whole-cell current (*I*_WC_; *top*) and unitary IPSC amplitude (*lower*) from the same CCK BC–CA1 PC pair over the course of the experiment, including the initial control conditions and following application of baclofen and then CGP to the bath. *Summary bar charts* of synaptic parameters for CCK BC–CA1 PC pairs (8 cells) under control conditions and during baclofen and CGP bath application: IPSC amplitude (**c**), apparent failures of transmission (**d**), paired-pulse ratio (**e**) and latency distribution (CV, **f**). **g–i** *Bar charts* of synaptic parameters for PV BC–CA1 PC pairs (11 cells) under control conditions, baclofen and CGP application: IPSC amplitude (**g**), failure rate of IPSCs (**h**) and the paired-pulse ratio (**i**). **j** *Summary bar chart* of the normalized IPSC amplitudes for a comparison of baclofen-induced inhibition at CCK and PV BC synapses. CGP recovery is shown for both cell types. Statistics shown: *ns P* > 0.05, * P < 0.05, ** P < 0.01, *** *P* < 0.001, results from repeated measures ANOVA with Sidak’s multiple comparison test or Mann–Whitney tests (**j**), all data is derived from 8 CCK BC/CA1 PC and 11 PV BC/CA1 PC pairs

Our previous recordings of synaptically coupled PV BC and PC pairs were consistent with the results of the minimal stimulation experiments and indicated an approximately 40% (of control) reduction in the unitary IPSCs during application of baclofen (10 µM; Booker et al. 2013, Fig. 7). To directly assess the effects of GABA_B_R activation on GABA release from CCK BCs, we performed paired recordings between identified CCK BCs and CA1 PCs (Fig. 2a). Interneurons were anatomically identified following overnight fixation, visualization of biocytin and immunolabeling with CCK antibodies. CCK BCs were identified based on the presence of dense axon in and near the *str. pyramidale*, forming ring like “baskets” around the somata of putative CA1 PCs (Fig. 2).

In dual recordings from eight synaptically coupled CCK BC to CA1 PC pairs, APs (2× with 50 ms interval) evoked in the CCK BCs were reliably followed by short-latency unitary IPSCs in the CA1 PCs (Fig. 2b, upper), with an average amplitude of 29.2 ± 9.3 pA of first IPSC and failure rate of 35.1 ± 8.9%. The second IPSCs showed marked paired-pulse depression (PPD) with a ratio of 0.47 ± 0.05. Consistent with a relatively low temporal precision of release at these synapses (Hefft and Jonas 2005), the coefficient of variation (CV) of the onset latency was 0.52 ± 0.12 (Fig. 2c–h). Bath application of 10 µM baclofen significantly reduced the average amplitude of the first IPSCs to 17.6 ± 6.1% to 4.8 ± 2.5 pA (*P* = 0.02, repeated measures 1-way ANOVA). In fact, the reduction here was comparable to that observed in the minimal stimulation experiments for WIN-sensitive afferents (*P* = 0.19, Mann–Whitney test). Application of CGP (5 µM) resulted in a full recovery of the IPSC amplitude (Fig. 2b, c). Consistent with a presynaptic locus of GABA_B_R activity, baclofen application resulted in an increase in failure rate to 76.6 ± 8.6% (*P* < 0.0001, repeated measures 1-way ANOVA) and enhanced PPD to 0.24 ± 0.08 (*P* = 0.02, repeated measures 1-way ANOVA). Furthermore, the CV of onset latency decreased to 0.32 ± 0.12 (*P* = 0.03, repeated measures 1-way ANOVA).

Given the apparent differences in the degree of presynaptic inhibition between CCK and PV BC-mediated transmission, we next performed paired recordings from PV BCs (4 cells) to further characterize these effects. PV BCs were all identified on the basis of PV immunoreactivity and axon collaterals localized to *str. pyramidale* (Supplementary Fig. 1) as previously described (Booker et al. 2013). Interestingly, this new sample included not only examples of BCs with vertically arranged dendrites spanning all layers, but also a horizontally oriented somato-dendritic domain confined to the *str. oriens* (Supplementary Fig. 1D). In all these PV BCs to CA1 PC paired recordings we observed a baclofen-produced inhibition of IPSC amplitude to 45.0 ± 5.1% of control levels, which recovered to control levels after the application of CGP (Fig. 2g). Thus, the presynaptic inhibitory effects in these pairs were markedly smaller than that observed for CCK BCs, but corresponded well to our previously published data (seven cells; 57.1 ± 0.4% of control; *P* = 0.49; Booker et al. 2013). As we had previously only analyzed the IPSC amplitude difference, we now reanalyzed these previously published cells for PPD and failure rate and included them with these four additional paired recordings (Fig. 2g–j). For the pooled PV BC data set (11 cells), GABA_B_R-mediated presynaptic inhibition reduced IPSC amplitude to 51.2 ± 6.0% of control levels (Fig. 2j), not different from that observed in M2R-sensitive synapses in Fig. 1 (*P* = 0.51, Mann–Whitney test), but reflecting a substantially lower inhibition than in CCK–BC pairs (*P* = 0.004, Mann–Whitney test). Analysis of IPSC failure rate showed that in baseline conditions PV BCs had less failures of transmission (22.4 ± 6.8%), which was not significantly different from that of CCK BCs (*P* = 0.39). However, during baclofen application PV BCs showed only a very modest increase in IPSC failure rate to 33.8 ± 9.4%, which was not different from control (*P* = 0.16, Wilcoxon signed rank test, Fig. 2h). Indeed, the failure rate of PV BC in baclofen was substantially lower than that of CCK BCs (*P* = 0.006, Mann–Whitney). Furthermore, as expected from the high release probability of PV BCs, they showed PPD (0.71 ± 0.05), which was removed by application of baclofen to 1.02 ± 0.1 (*P* = 0.02, 1-way ANOVA with multiple comparisons). Both the effect on IPSC amplitude and failure rate in PV BCs was reversed by subsequent application of CGP. Interestingly, unlike CCK BCs, CGP in PV BCs application resulted in a stronger PPD than under control conditions (0.54 ± 0.6, *P* = 0.025) suggesting that under control conditions there might be a level of tonic effect of GABA_B_R on presynaptic short-term plasticity. These data show that despite both populations of BCs express presynaptic GABA_B_Rs, the ability of these receptors to inhibit GABA release onto CA1 PC somata is substantially different. Furthermore, CCK and PV BC synapses onto CA1 PCs show divergence in the effects of GABA_B_R activation on short-term plasticity and the failures of transmission.

**Fig. 3:**
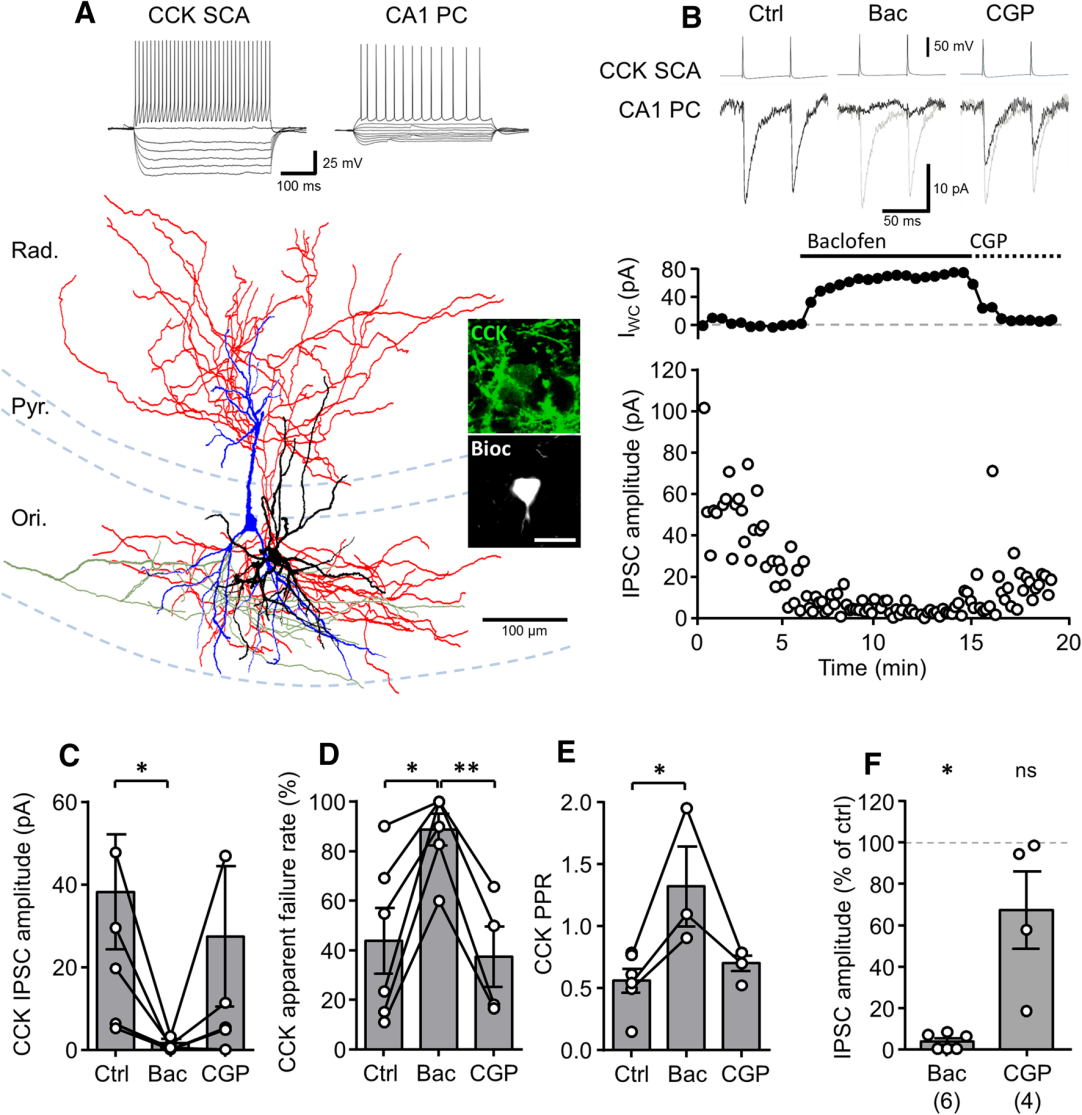
Identified CCK SCA cells show strong presynaptic GABA_B_R-mediated inhibition. **a** Reconstruction of a synaptically coupled CCK SCA (soma and dendrites in *black*, axon in *red*) and a CA1 PC (soma and dendrites in *blue*, axon in *green*). *Inset* (*top*), voltage responses elicited in the CCK SCA cells and the CA1 PCs to hyper- to depolarizing current steps (−500 to 500 pA, 50 pA steps, 500 ms duration). *Inset* (*right*), immunolabeling for CCK (in *green pseudocolor*) in the soma of the SCA cell (in *gray*). Pyr., *stratum pyramidale*; Ori., *stratum oriens*; Rad., *stratum radiatum*. **b**, *upper*, Representative pairs of action potentials elicited in the SCA cell shown in **a** (*upper traces*, suprathreshold current pulses of 1–2 nA, 1 ms duration, 50 ms interval) were followed by short-latency IPSCs in the recorded CA1 PC (*lower traces*) under control conditions (*left panel*), during bath application of baclofen (10 µM, *middle panel*), and CGP (10 µM; *right panel*); baclofen and CGP are underlain by control traces (*gray*). **b**, *lower*, time-course plot of the postsynaptic whole-cell current (*I*_WC_; *top*) and unitary IPSC amplitude (*lower*) from the same CCK SCA cell–CA1 PC pair during the experiment, including control period, and subsequent baclofen and CGP applications. *Summary bar charts* of synaptic properties of CCK SCA–CA1 PC pairs (six pairs) during each pharmacological epoch: IPSC amplitude (**c**), apparent failures of transmission (**d**) and paired-pulse ratio (PPR, **e**). **f** Summary bar chart of the normalized IPSC amplitudes during baclofen and CGP applications in CCK SCA cells. Statistics shown: *ns P* > 0.05, * P < 0.05, ** *P* < 0.01, results from Wilcoxon signed rank tests, all data are derived from 6 CCK SCA–CA1 PC pairs

In a subset of paired recordings, we found that the presynaptic CCK IN had axon collaterals in *str. radiatum* and *oriens*, targeting dendritic domains of CA1 PCs, consistent with the Schaffer-Collateral Associated (SCA) type (Booker et al. 2017). It is known that CCK SCA cells share many of the same presynaptic properties of CCK BCs, including CB1R sensitivity (Lee and Soltesz 2011), so we next asked if CCK SCA cells also showed presynaptic GABA_B_R-mediated inhibition. In the six synaptically coupled SCA to CA1 PC pairs (Fig. 3) we observed IPSCs in response to presynaptic APs with an average amplitude of 9.1 ± 7.0 pA (first IPSCs), which was reduced to 3.5 ± 1.5% of control following baclofen application (Fig. 3b, c, f), similar to that observed in CCK BCs (*P* = 0.13, Mann–Whitney test). IPSC amplitudes recovered to close to control levels with application of CGP (*P* = 0.06, Wilcoxon signed rank test). CCK SCA cells had a failure rate of 44.1 ± 13.2%, which increased to 88.8 ± 6.4% upon baclofen application (*P* = 0.03, Wilcoxon signed rank test, Fig. 3d). They showed a PPD of 0.56 ± 0.1, similar to that of CCK BCs (*P* = 0.28, Mann–Whitney test), but unlike CCK BCs, in SCA cells the PPD changed to facilitation with a ratio of 1.33 ± 0.32 upon application of baclofen (*P* = 0.02, Mann–Whitney test, Fig. 3e). However, in three SCA cells paired-pulse ratio could not be measured following baclofen application due to complete inhibition of the IPSC. Together, these data suggest that dendritic inhibitory CCK SCA cells possess similar presynaptic GABA_B_R inhibition to their BC counterparts, with potential divergence in its effect on short-term plasticity.

### Different surface densities of presynaptic GABA_B_Rs at perisomatic synapses

**Fig. 4:**
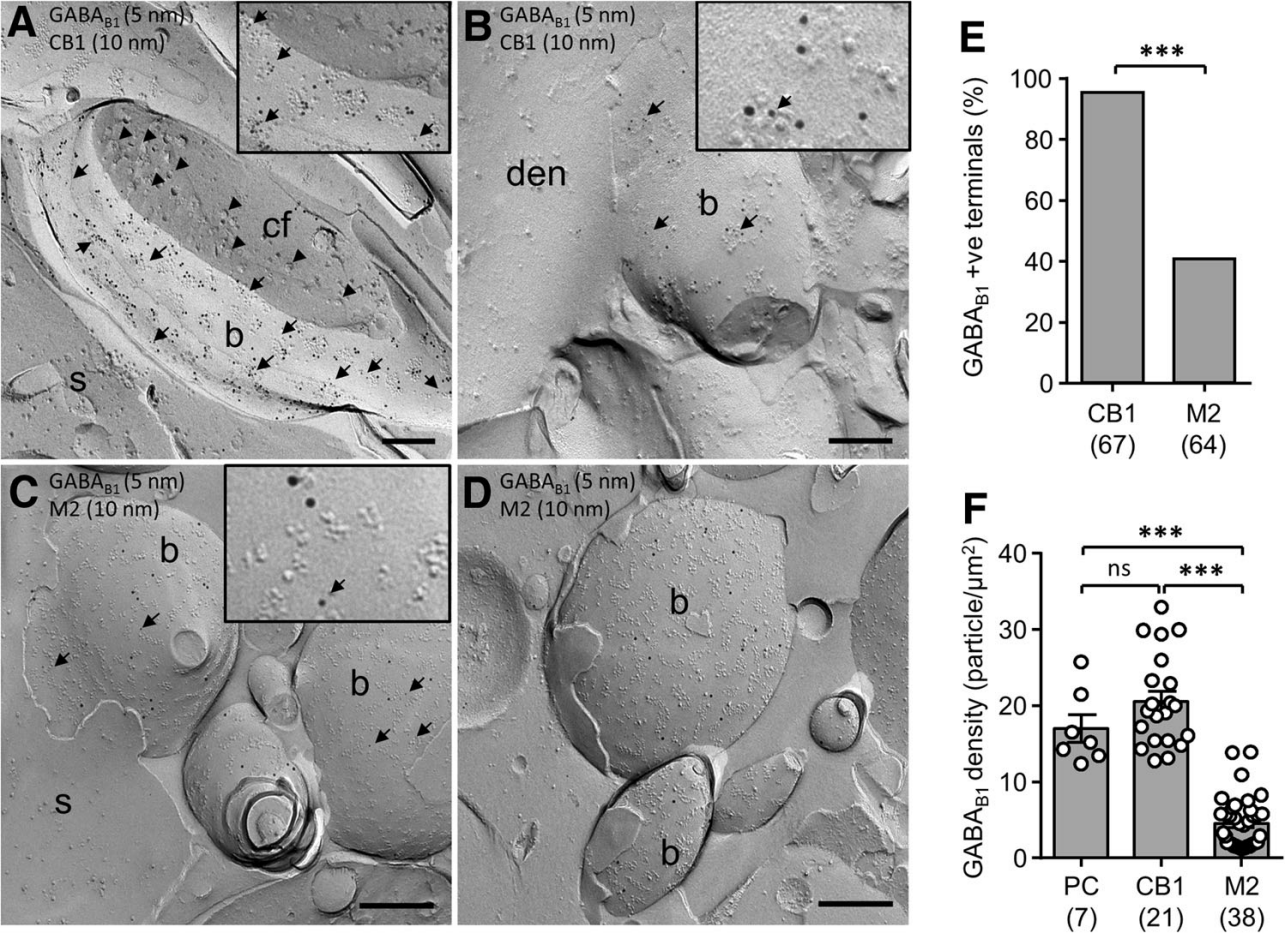
Surface expression of GABA_B1_ differs on CB1R- and M2R-positive axon terminals. **a**, **b** Electron micrographs of CB1R-positive (10 nm gold particles) putative CCK BC axon terminals (*b*) forming synapses either with somata (*S* in A) or proximal dendritic shafts (*den* in B) of putative CA1 PCs. Note, that CB1R-positive terminals show consistently high levels of immunogold labeling for GABA_B1_ (5 nm, *arrows*) (synaptic vesicles are indicated by *arrowheads* in **a**). **c**, **d** Electron micrographs of M2R-positive (10 nm immunogold) putative PV BC axon terminals (*b*) contacting somata (*S*) of putative CA1 PCs. M2R-positive terminals showed a lower GABA_B1_ density (5 nm particles, *arrows* in **c**) and also included immunonegative terminals (*b* in **d**). *Insets* in **a**–**c** show differences in particle size, with small 5 nm gold particles for GABA_B1_ (*arrows*) and the larger 10 nm particles for the interneuron-specific marker CB1R and M2R, respectively. **e** Summary bar chart of the proportion of GABA_B1_ on double-labeled CB1R- or M2R-containing axon terminals. The total number of examined CB1R- and M2R-immunopositive terminals is indicated in parenthesis. **f** Quantification of the surface density of immunogold particles for GABA_B1_ subunit on CB1R- and M2R-positive axon terminals (with GABA_B1_ immuno-negative profiles exclude) in comparison to PC dendrites. Note the divergence of labeling density between CB1R- and M2R-positive terminals. Statistics shown: *ns P* > 0.05, *** P < 0.0001, Fisher’s exact test and 1-way ANOVA with multiple comparisons. *Scale bars* 200 nm

To determine whether the differences in GABA_B_R-mediated presynaptic inhibition of BC terminals are due to differential distribution pattern and/or density of functional receptors we performed quantitative SDS-FRL electron microscopy. Immunolabeling for the two marker receptors, CB1R (Fig. 4a, b) and M2R (Fig. 4c, d), showed characteristic labeling of putative presynaptic axon terminals in *str. pyramidale* of CA1. Labeling for CB1R was consistently stronger on a subset of perisomatic terminals, while M2R-positive terminals showed generally weaker labeling. When GABA_B1_ subunit localization was examined on CB1R-positive terminals it was found that nearly all terminals contained immunogold particles for GABA_B1_ (95.2% of terminals, 67 terminals) (Fig. 4a, b), whereas only 40.6% of M2R-positive terminals (64 terminals) showed immunoreactivity for GABA_B1_ (Fig. 4c, d), which was significantly lower than on CB1R-positive terminals (*P* < 0.0001, Fisher exact test, Fig. 4e). We then asked whether there was also a difference in the density of the GABA_B1_ receptor subunit on terminals. In a subset of CB1R-positive terminals, which all showed GABA_B1_ labeling (23 terminals), GABA_B1_ surface density was high at 20.6 ± 1.3 particles/µm^2^. This density was similarly high to that of CA1 PC dendrites in *str. radiatum* (17.0 ± 1.8 particles/µm^2^, 7 dendrites; *P* = 0.23, 1-way ANOVA with multiple comparisons). Interestingly, on M2R-containing axon terminals we observed a much weaker GABA_B1_ labeling density at 4.5 ± 0.5 particles/µm^2^ (38 axon terminals), which was substantially and significantly lower than that of both CB1R-containing terminals and CA1 PC dendrites (both *P* < 0.0001, 1-way ANOVA with multiple comparisons, Fig. 4f).

These data confirm that there are IN type-specific differences in the presynaptic GABA_B_R expression in inhibitory axon terminals, as measured by the density of the constitutive subunit GABA_B1_, between putative CCK and PV BC. Interestingly, this data shows that not only is the number of terminals that containing GABA_B1_ different, but also the receptor density on those terminals is different, plausibly explaining the differences we observed in GABA_B_R-mediated presynaptic inhibition between CB1R- and M2R-containing synapses.

## Discussion

Presynaptic neuromodulation is a central neuronal property, which defines cell types as well as our understanding of the role those cells play in local networks. Our results demonstrate that while both CCK and PV BCs contain presynaptic GABA_B_Rs at their axon terminals, acting as autoreceptors, their surface density and inhibitory potential at these two synapses show marked differences. We provide compelling evidence that virtually all CCK BC terminals contain GABA_B_Rs at high levels and GABA release is dramatically inhibited by their activation. In contrast, only 40% of PV BC axon terminals express GABA_B_Rs at detectable levels, leading to only a moderate reduction in inhibitory transmission at these output synapses upon GABA_B_R activation. These findings add to an increasing list of molecular and functional differences between these two major IN types with implications for their network function.

### Presynaptic GABA_B_Rs as auto- and heteroreceptors

Presynaptic control of synaptic transmission by GABA_B_Rs has been established for various transmitter systems, including glutamatergic and GABAergic transmission (Bowery et al. 1981; Connors et al. 1988; Deisz and Prince 1989). Accordingly, the robust inhibitory effect of GABA_B_Rs as presynaptic autoreceptors at GABAergic synapses has been well documented in diverse brain regions including the neocortex and hippocampus (Misgeld et al. 1984; Peet and McLennan 1986; Oláh et al. 2009). However, while divergence in the sensitivity to GABA_B_R-mediated heterosynaptic inhibition at major glutamatergic pathways have been long recognized in the forebrain (Lanthorn and Cotman 1981; Ault and Nadler 1983; Molyneaux and Hasselmo 2002), little is known of GABA_B_Rs in diverse INs types, let alone the differential nature of this. Prior in situ and immunocytochemical studies suggested that GABA_B_Rs are present at high levels in somata of some, but not all interneuron types (Fritschy et al. 1999; Sloviter et al. 1999). In fact, for postsynaptic compartments our recent results demonstrated that morphological subtypes of PV and CCK INs show substantial differences in GABA_B_R surface density, as well as in the postsynaptic currents (Booker et al. 2013, 2017). Furthermore, at the presynaptic level, dendritic- and perisomatic-targeting PV IN subtypes were found to be sensitive to GABA_B_R-mediated inhibition at their output synapses, albeit differentially (Booker et al. 2013). For perisomatic synapses formed by putative BCs, earlier electrophysiological investigations using minimal stimulation indicated that GABA_B_Rs modulate GABA release at most, but not all axon terminals (Lambert and Wilson 1993). Consistent with these findings the present anatomical results show that individual perisomatic inhibitory axon terminals contain variable levels of the receptor depending on their neurochemical identity: CCK-positive terminals have consistently high densities, whereas PV-positive axon terminals show a dichotomy with one half of them containing an approximately four-times lower density and the other half being negative for GABA_B_Rs. In good agreement, our data obtained by minimally stimulation and paired recordings converges, demonstrating that GABA_B_R activation markedly reduces transmission at CCK output synapses (Neu et al. 2007; Lee and Soltesz 2011; Booker et al. 2013; Jappy et al. 2016), but has an approximately twofold weaker effect at PV synapses at the concentration of baclofen used in our experiments. In contrast to the findings of Lambert and Wilson (1993), however, all putative PV axons and identified unitary connections were affected to a similar degree and no baclofen insensitive connections were found. These electrophysiological findings suggest that the molecular dichotomy observed at the level of the individual axon terminals in the electron microscopic level (see Fig. 4) affects the multiple synaptic contacts formed between individual pre- and postsynaptic neurons (Buhl et al. 1994; Bartos et al. 2001) in a stochastic manner. What trafficking mechanisms can maintain such a homogeneous distribution of dichotomy in individual axons remains an interesting open question.

### Molecular and functional dichotomy of BC synapses

GABAergic INs show a high level of diversity, plausibly reflecting their functional specification in cortical circuits. In good agreement with this concept, the two BC types show striking differences in their anatomical, physiological and molecular properties (Freund and Katona 2007; Armstrong and Soltesz 2012). Axon terminals of CCK and PV BCs differ in their complementary expression of VGCCs at the active zone (Ca_v_2.2 versus Ca_v_2.1 types, respectively) and presynaptic neuromodulatory receptors (CB1R versus M2R and µ-opioid receptors, respectively) (Hefft and Jonas 2005; Neu et al. 2007; Lee and Soltesz 2011; Jappy et al. 2016). While both CCK and PV BCs express GABA_B_ autoreceptors at their output synapses (Neu et al. 2007; Lee and Soltesz 2011; Booker et al. 2013), our results now demonstrate that their expression and functional impact is quantitatively different between the two types. The strong presynaptic inhibitory action mediated by GABA_B_Rs at CCK synapses is consistent with previous findings that transmission is reduced by 70–100% in the presence of baclofen in a concentration range of 1–100 µM (Neu et al. 2007; Lee and Soltesz 2011; Jappy et al. 2016). This strong presynaptic autocrine modulation converges with the similarly strong retrograde control, exerted by the endocannabinoids system via CB1R (Katona et al. 1999; Neu et al. 2007; Lee and Soltesz 2011; Jappy et al. 2016). The effect of GABA_B_R activation at PV BC synapses, in contrast, is substantially smaller; however, our data is not consistent with a general lack of GABA_B_R at these synapses as assumed earlier (Freund and Katona 2007).

In addition to the changes in the amplitude and reliability of ISPCs, baclofen has differential effects on the dynamic behavior of CCK and PV synapses: PPD observed at PV output synapses was fully abolished by the drug and recovered by subsequent application of the selective antagonist CGP. This finding is in good agreement with previous observations that GABA_B_R activation eliminates short-term depression at both excitatory and inhibitory synapses in the hippocampus and can thereby maintain synaptic transmission during repeated activation albeit at reduced amplitudes (Lei and McBain 2003). Surprisingly, PPD observed at CCK BC synapses was not reduced but enhanced in the presence of baclofen, which also recovered to control levels in CGP. These results are incompatible with a tonic control of perisomatic inhibitory transmission or a direct involvement of these receptors in frequency dependent behavior at the time scales investigated. Rather, our observations plausibly reflect indirect effects due to the modulation of release machinery and the probability of GABA release (Dittman et al. 2000; Lei and McBain 2003).

Finally, perisomatic CCK synapses differ in their long-term plasticity: while theta-burst stimulation induces typically LTP at inhibitory synapses, the output synapses of CCK interneurons display LTD. Although the induction of LTD requires activation of postsynaptic GABA_B_Rs with an inhibitory effect on adenyl cyclase and PKA activity (Jappy et al. 2016), it remains open what is the contribution of the highly expressed presynaptic receptors.

### Functional implications of the dichotomy of perisomatic synapses

While the two BC types converge on the perisomatic compartment of their postsynaptic targets, their distinct properties and activity patterns in vitro and in vivo reflect distinct functions in the network. A differential contribution of the two types to network activity patterns is well established (Klausberger and Somogyi 2008): CCK BCs integrate incoming inputs over longer time scales and discharge and release GABA with low temporal precision, mediating a tonic form of inhibition to their targets (Hefft and Jonas 2005; Daw et al. 2009; Cea-del Rio et al. 2011). Activation of presynaptic GABA_B_Rs could therefore substantially suppress CCK BC output, resulting in dynamic disinhibition of principal cells, enhanced network activity and facilitation of long-term potentiation among principal cells (Davies et al. 1991; Mott and Lewis 1991). In contrast, PV BCs are proposed to act as fast-signaling devices and contribute to the generation of fast oscillations at gamma and ripple frequencies (Jonas et al. 2004; Bartos et al. 2007; Sohal et al. 2009). Enhanced GABA_B_R-mediated presynaptic inhibition at PV BC synapses during network activity (Scanziani 2000) may dampen, but will not abolish the entrainment of target cell populations, due to its moderate effect on IPSC amplitudes and the removal of PPD.

Beyond these physiological network functions, evidence is mounting for significant role for presynaptic GABA_B_Rs in the aetiology of neuropathological disorders, notably epilepsy and intellectual disabilities (i.e. Fragile X syndrome). In epilepsy, while a selective decrease of CCK BC terminals have been observed in the CA1 (Wyeth et al. 2010), GABA_B_Rs expression at CCK axon terminals appears to be increased leading to stronger disinhibitory effects of principal cells potentially promoting seizure generation and the progression of epilepsy (Dugladze et al. 2013). Interestingly, a similar hypersensitivity of GABA_B_R-mediated presynaptic inhibition of feedforward inhibition was observed in a mouse model of Fragile X syndrome (Wahlstrom-Helgren and Klyachko 2015). While, the IN subtype involved was not identified in these experiments, neurogliaform and CCK interneurons are likely candidates due to their high expression of the receptors. The increased presynaptic sensitivity leads to an altered excitation/inhibition balance in the network, which is thought to be an underlying circuit mechanism of cognitive impairments observed both in patients and animal models (Silverman et al. 2015; Wahlstrom-Helgren and Klyachko 2015).

In conclusion, we provide the first direct comparative and quantitative data showing that presynaptic GABA_B_Rs exert differential inhibition at CCK and PV BC synapses, resulting from a higher surface expression of the receptor at all CCK BC terminals and a lower protein density present in a subpopulation of boutons of PV BCs. These data provide further evidence that distinguishes these two major IN types with respect to their presynaptic molecular composition and physiological functions.

## Electronic supplementary material

Below is the link to the electronic supplementary material.

Supplementary material 1 (DOC 1243 kb)
