# Supplementary material for: Differential surface density and modulatory effects of presynaptic GABAB receptors in hippocampal cholecystokinin and parvalbumin basket cells
Source: Brain Struct Funct. 2017 May 2;222(8):3677–90. doi: 10.1007/s00429-017-1427-x (PMC5676818; doi:10.1007/s00429-017-1427-x)
Supplement: Supplementary file 1 — Supplementary material 1 (DOC 1243 kb) [file 429_2017_1427_MOESM1_ESM.doc]

**Supplementary Material** - Booker *et al.,* (2017) Brain Structure and Function.


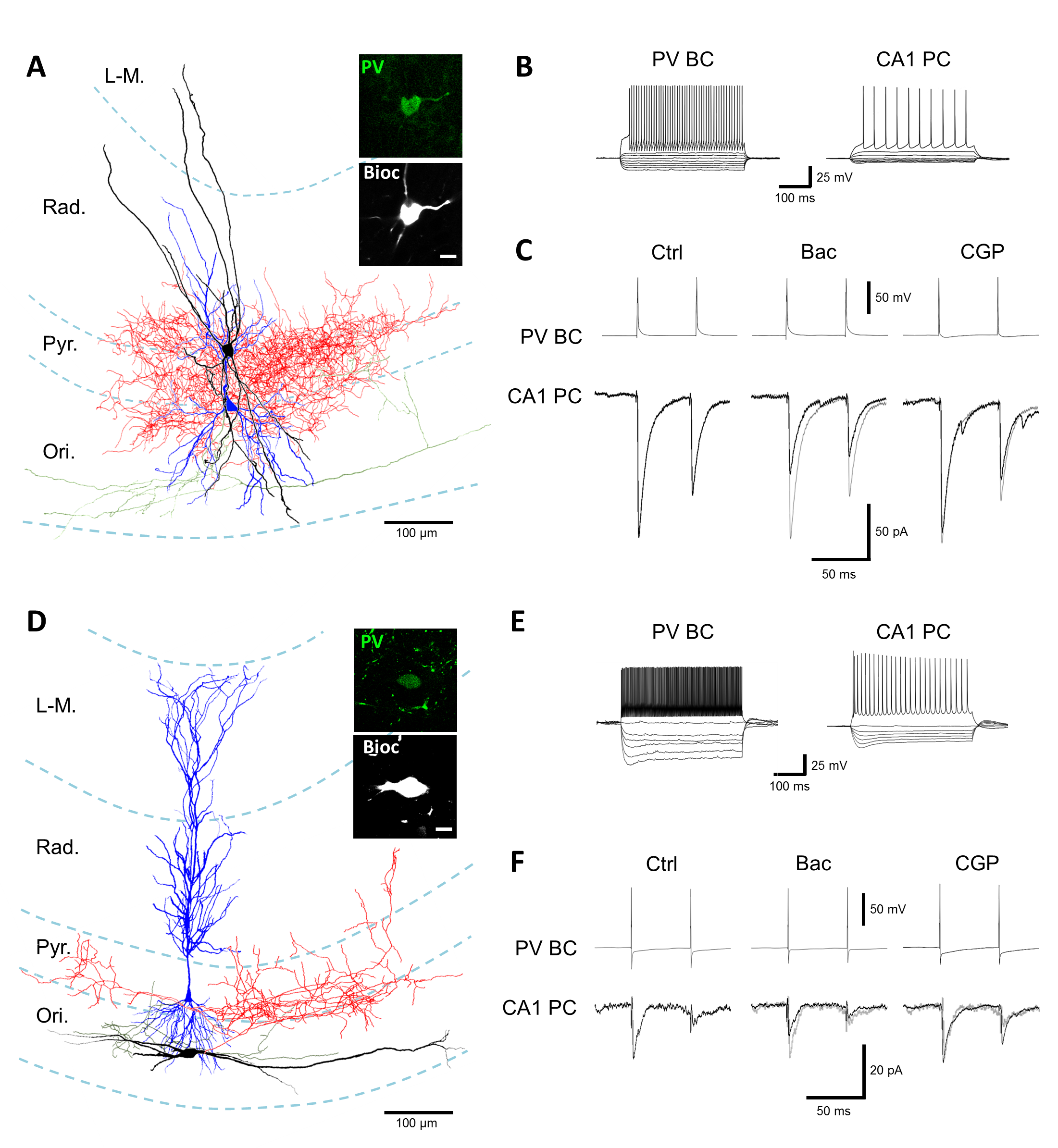


**Supplementary Figure 1:** GABA_B_Rs inhibit GABA release from morphologically diverse PV BCs. **A**, Morphological reconstruction of a synaptically-coupled PV BC (soma and dendrites in black, axon in red) and CA1 PC (soma and dendrites in blue; axon in green) pair. The BC has a characteristic morphology with vertically oriented dendrites spanning all layers and a dense axon in and near the *stratum pyramidale* (Pyr.). Inset, immunoreactivity for PV (green pseudocolor) in the biocytin-filled soma of the BC (grayscale). Abbreviations: Ori., *stratum oriens*; Rad.*, stratum radiatum*; L-M, *stratum lacunosum-moleculare*. **B,** Voltage responses of the PV BC and CA1 PC pair to hyper- to depolarizing current pulses (-500 to 500 pA, 50pA steps, 500ms duration). **C,** Pairs of action potentials elicited in the same PV BC (upper traces, current pulses of 1-2 nA, 1 ms duration, 50 ms interval) were followed with short latency unitary IPSCs in the postsynaptic CA1 PC (lower traces) under control conditions (left panel) and during sequential bath application of baclofen (10 µM, middle panel) and CGP (10 µM; right panel); baclofen and CGP traces are underlain by the control trace for comparison (in grey). Note the near 50% reduction in IPSC amplitude following baclofen application. **D – F,** Layout according to the same scheme as in **A – C** for a horizontal PV BC with dendrites confined to *str. oriens*. Note the comparable, approximately 50% reduction in the IPSC amplitude in the synaptically-coupled PC during baclofen application indicating that the presynaptic effect of GABA_B_R activation is consistent amongst BCs despite divergent morphologies.
